# Acute lower respiratory tract infection due to respiratory syncytial virus in a group of Egyptian children under 5 years of age

**DOI:** 10.1186/1824-7288-37-14

**Published:** 2011-04-06

**Authors:** Aya M Fattouh, Yasmeen A Mansi, Mervat G El-anany, Amany A El-kholy, Hanaa M El-karaksy

**Affiliations:** 1 Department of Pediatrics, Faculty of Medicine, Cairo University, Cairo, Egypt; 2 Department of Clinical Pathology, Faculty of Medicine, Cairo University, Cairo, Egypt

## Abstract

**Background and aim:**

Respiratory syncytial virus (RSV) is one of the most important causes of acute lower respiratory tract infections (ALRTI) in infants and young children. This study was conducted to describe the epidemiology of ALRTI associated with RSV among children ≤ 5 years old in Egypt.

**Patients and Methods:**

We enrolled 427 children ≤ 5 years old diagnosed with ALRTI attending the outpatient clinic or Emergency Department (ED) of Children Hospital, Cairo University during a one- year period. Nasopharyngeal aspirates were obtained from the patients, kept on ice and processed within 2 hours of collection. Immunoflourescent assay (IFA) for RSV was performed.

**Results:**

91 cases (21.3%) had viral etiology with RSV antigens detected in 70 cases (16.4%). The RSV positive cases were significantly younger than other non-RSV cases (mean age 8.2 months versus 14.2 months, p <0.001). RSV cases had significantly higher respiratory rate in the age group between 2-11 months (mean 58.4 versus 52.7/minute, p < 0.001) and no significant difference in the mean respiratory rate in the age group between 12-59 months. More RSV cases required supplemental oxygen (46% versus 23.5%, p < 0.001) with higher rate of hospitalization (37.1% versus 11.2%, p < 0.001) than the non-RSV cases. 97% of RSV cases occurred in winter season (p < 0.001).

**Conclusion:**

RSV is the most common viral etiology of ALRTI in children below 5 years of age, especially in young infants below 6 months of age. It is more prevalent in winter and tends to cause severe infection.

## Introduction

Viral infections are the most frequent etiological agents for acute respiratory infections, and are responsible for a significant morbidity and mortality in children [1].

Respiratory syncytial virus (RSV) is well recognized as the most important pathogen causing acute respiratory disease in infants and young children, mainly in the form of bronchiolitis and pneumonia [2]. Influenza viruses' type A and B (FLU A/B), parainfluenza virus (PIV), adenovirus (ADV), and human metapneumovirus (hMPV) are other important viral etiologic agents of ALRTI [1].

RSV infection is associated with significant disease burden in infants and young children in terms of hospitalization, related complications, and even mortality[3]. Identification of the etiologic agents of ALRTI and monitoring their trends, in a particular setting, improve patient management, and guide antimicrobial utilization and implementation of infection control precautions. The epidemiology of RSV has not been studied in Egypt before.

The objective of this study was to describe the epidemiology and clinical characteristics of ALRTI caused by RSV among a group of Egyptian children below 5 years of age and to compare between children infected with RSV and those that had ALRTI but did not have RSV.

## Materials & methods

### Patients

We recruited four hundred and twenty seven children (427) less than five years of age presenting with ALRTI from the out-patient clinic and emergency department of Cairo University Children Hospitals over one year between December 2006 and November 2007. We obtained the pediatric department research review board approval.

A case of pneumonia was defined; depending on WHO definitions, as a child with cough and difficult breathing or tachypnea (50 breaths per minute or more in a child aged 2-11 months or 40 breaths per minute or more in a child aged 12-59 months) and if the patient exhibit lower chest in-drawing or stridor, he will be diagnosed as a case of severe pneumonia [4]. Those who are not fast breathing was diagnosed as a case of bronchitis or wheezing bronchitis. Patients; included depending on clinical findings, were eligible for inclusion whether they were managed as outpatients or were hospitalized but those with symptoms longer than four weeks were excluded.

Following informed parent consent, patients were subjected to a questionnaire detailing demographic data and past medical history completed by the parents/guardians.

Full clinical history was obtained including the current illness with special emphasis on the presence of extra- pulmonary manifestations as inability to feed or drink, vomiting, diarrhea, convulsions, altered mental status or conjunctivitis. The history included data about any received antibiotic therapy, previous episodes diagnosed as lower respiratory tract infection and any associated conditions especially congenital heart disease, asthma, malnutrition, renal disorders or diabetes. Accurate gestational age of the included patients could not be obtained as those patients were selected from the outpatients' clinic and the ED and their parents did not have any birth related documents.

Physical examination included general examination and thorough chest examination to detect the presence of respiratory distress (tachypnea, chest indrawing, grunting or cyanosis) and other chest findings as wheezing and crepitations. Chest x-ray and complete blood count were done for those patients whose conditions required hospital admission.

### Specimens

Nasopharyngeal aspirate samples were collected in sterile vials from each enrolled child. The specimens were collected between 1--15 days of illness. The sample was kept on ice and processed within 2 hours of collection [5]. Immunoflourescent assay (IFA) for RSV, adenovirus, influenza A virus, influenza B virus, and parainfluenza 1-3 viruses (Panel I Viral Screening and Identification Kit, Chemicon International, Inc., Temecula, CA) was performed on each sample.

Blood was collected aseptically using venipuncture techniques and serum samples were refrigerated at -70°C. Indirect immuno-enzyme assay (ELISA) was used to test for IgG and IgM of *Chlamydophila *and *Mycoplasma *using VIRCELL kit (VIRCELL, S.L. Pza.Dominguez Ortiz 1.Poligono Industrial Dos de October 18320 Santa Fe, Granada, Spain; lot number 06ECPN107).

Clinical management and outcome data were collected.

### Statistical analysis

Data were statistically described in terms of mean ± standard deviation (± SD), frequencies (number of cases) and percentages when appropriate. Comparison of quantitative variables between the study groups was done using Student *t *test for independent samples. For comparing categorical data, Chi square (χ^2^) test was performed. Exact test was used instead when the expected frequency is less than 5. A probability value (*p *value) less than 0.05 was considered statistically significant. All statistical calculations were done using computer programs Microsoft Excel 2003 (Microsoft Corporation, NY, and USA) and SPSS (Statistical Package for the Social Science; SPSS Inc., Chicago, IL, USA) version 15 for Microsoft Windows.

## Results

A viral agent was detected in 91 children (21.3%) of the enrolled cases, 70 children of them (16.4%) were infected with RSV. The other viruses that were identified included: para-influenza 3 virus in 14 children (3.3%); influenza A virus in 3 children (0.7%), adenovirus in 2 and para-influenza 1 in 2 cases (0.5%). There was no overlap in infection between the RSV virus and the other identified viruses.

Among the RSV cases, 34 (48.6%) were males. Their median age was 3 months (range between 8 days and 4 years) and 45 cases (64%) of them were less than 6 months. Fifty one children (72.8%) had received antibiotic therapy for a median of 3 days (range between 1-13 days) before admission. Regarding the clinical diagnosis of the RSV cases, 43 patients were diagnosed as pneumonia (61.4%) while 22 cases were diagnosed as bronchitis (31.4%) and 5 cases with wheezing bronchitis (7.1%) Twenty five cases (35.7%) reported having a pre-existing medical condition; 14 cases (21.2%) were small for weight, 5 cases (7.1%) had recurrent wheezy chest, 4 cases (5.7%) suffered from a congenital heart disease, one patient with chronic renal failure and one with diabetes. They did not have different outcomes.

Comparing the RSV cases and the remaining 357 patients showed significantly lower mean age of the RSV cases. The mean weight of the RSV cases was significantly lower than the non RSV cases but actually the non RSV cases with small weight for age were significantly more than the RSV cases (21.2% versus 37.8%, p value 0.024). RSV infection was associated with significantly higher respiratory rates in the age group between 2-11 months (58.4 ± 9.2 versus 52.7 ± 9.3, p <0.001) while in those between 12-59 months although showed higher respiratory rate but with no significant difference (47.7 ± 8.53 versus 46.8 ± 8.48, p 0.724). Chest in-drawing was significantly more among the RSV cases. The rate of hospitalization was significantly higher among RSV cases (26/70 cases 37.1%, compared to 40/357 non-RSV cases 11.2%) with median days of hospitalization 3 days (range 1 - 15). Also, significantly more RSV cases required supplemental oxygen (32/70 compared to 84/357). Only one patient died in each group and this leads to a significantly higher case fatality rate among the RSV cases (1.4% versus 0.3%, p < 0.001, Odds ratio 5.159, CI 95%= 0.319 -83.483) (Table 1). These two patients had no underlying diseases or risk factors

**Table 1:** The clinical characteristics of the RSV vs. non RSV cases

	RSV (n = 70)	Non-RSV (n = 357)	P-value
Mean Age ± std. deviation (months)	8.2 (± 11.5)	14.2 (± 14.37)	0.001

Mean Weight ± std. deviation (kg)	6.7(± 3.3)	8.5(± 5.4)	0.006

Patients with small weight for age (%)	21.2%	37.8%	0.024

Mean Respiratory rate ± std. deviation (/min) in patients 2-11 mo	58.4 (± 9.2)	52.7 (± 9.3)	<0.001

Mean Respiratory rate ± std. deviation (/min) in patients 12-59 mo	47.7(± 8.53)	46.8(± 8.48)	0.724

Tachypnea (%)	62.9%	54.9%	0.137

Wheezes (%)	7.1%	9.8%	0.635

Difficult breathing (%)	98	91	0.053

Chest in-drawing (%)	88	74	0.014

Cough (%)	100	97	0.18

Inability to feed (%)	52	44	0.13

Convulsions (%)	1.7	3.3	0.4

Past history of pneumonia (%)	14	31	0.003

Supplemental Oxygen (%)	46	23.5	<0.001

Hospitalization (%)	37.1	11.2	<0.001

Admission to ICU (%)	1.5	1.1	0.59

Case fatality rate (%)	1.4	0.3	<0.001

**Figure 1:**
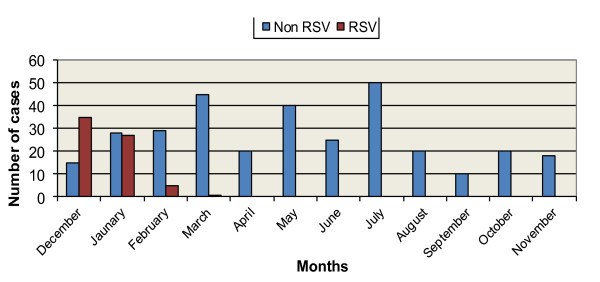
**shows the monthly distribution of RSV cases compared to the other cases**.

On analyzing the data about seasonal distribution of the RSV cases and the non RSV cases; we found that 68 children (97.1%) of the RSV cases presented during the 3-month-period from December to February. For the two months of December and January, RSV was the etiology for 59% of the enrolled ALRTI cases (Figure 1).

## Discussion

In the present study, the overall incidence of viral infection defined by IFA alone on nasopharyngeal specimens was 21%. The most frequently encountered virus was RSV (16.4%). The reported rates of RSV in other series were generally comparable to that reported in the present study [6-11]. Other studies have used different methods of viral detection as real-time PCR which resulted in higher rates of RSV detection up to 44-49% [12,13]. The different inclusion criteria in the different series contributed to the different rates of RSV detection as in our study we included only patients less than 5 years old who presented with ALRTI while other studies have included wider range of clinical diagnosis such as upper respiratory tract infection and even isolated fever resulting in higher rate of viral detection [14]. Others who focused on patients presenting with bronchiolitis or asthmatic bronchiolitis reported even higher rates of RSV detection reaching up to 56-72% (8&15). This may be attributed to the strong association between RSV infection early in life and the subsequent development of asthma [16]. The mechanism by which RSV contributes to asthma is complex and remains largely unknown although RSV-infected patients have increased levels of Th2 cytokines and IgE in the patients' sera, suggesting that an allergy-like condition may develop during infection [17]. In our study, 7.1% of RSV cases were admitted with the diagnosis of wheezing bronchitis and gave history or recurrent wheezy chest.

The RSV cases had significantly lower age mean than the non-RSV cases with 64% of them younger than 6 months old. The young age has been documented as a risk factor for RSV infection in many studies [9,10,14,17]. This can be attributed to the lower cellular immunity [10]. At the same time, this is an age when maternally acquired antibodies are decreasing with a half-life of about one month associated with lower magnitude of the humoral immune response to RSV in children <3 months [18].

In our study, we included patients with ALRTI so cough was a persistent feature in all patients while difficult breathing was encountered in almost all patients. We tried to demonstrate any useful association to predict RSV infection from clinical data and we found that RSV patients demonstrated higher respiratory rates, especially in the age group 2-11 months which includes 80% of the RSV cases; this was also observed by other investigators [19]. Chest in-drawing was encountered more frequent in RSV patients. These findings are consistent with Durain et al. who concluded that cough and retractions are predictive signals of RSV infection in young infants [20].

Another important feature of RSV infection is the seasonality of RSV infection. In temperate climate, RSV activity increases in the winter months but may occur year around in equatorial areas [21-23] while in tropical and subtropical areas, RSV infection peaks more often in relation to wet season [24]. In the current study, most RSV cases (97.1% of cases) occurred mainly between December and February; which are the cold months of the year in Egypt. In other countries like Italy, the peak of the RSV epidemic occurs in February, with an earlier occurrence and disappearance in the northern and central regions, compared to the southern regions [25]. The study of the local epidemiology of RSV infection is essential for predicting epidemics and planning for preventive measures especially for the high risk groups.

RSV infection in our study tended to be severe as demonstrated by observing the higher hospitalization rate (3 times greater than other etiologies, p < 0.001) which is consistent with other series [25-27] and the greater need to supplemental oxygen. Although the case fatality rate was significantly higher among the RSV cases but this may be due to the fact that the number of deaths are too small to make a clinically significant comparison.

There are some well-known medical risk factors associated with RSV infection as prematurity, chronic lung disease and congenital heart disease. Congenital heart disease had been encountered in 5.7% of our RSV cases. One of the limitations of the current study was the inability to investigate the other risk factors especially prematurity due to the fact that our patients were selected from the outpatient clinic or the ED with lack of accurate documented information about their gestational age. We couldn't rely on the data given by their parents which could have interfered with the accuracy of our results.

There are other host environmental factors such as severe stunting which was found to be associated with increased risk of progression to RSV associated pneumonia [28]. In our study, the mean weight in RSV cases was much lower than other patients but this was attributed to the lower mean age of the RSV cases.

One of the limitations in the present study was that we did not investigate the bacterial infection in depth to accurately identify the need for antibiotic administration. But we cannot ignore the fact that the majority of RSV cases (72.8%) have received antimicrobials prior to identification of a viral etiology and the same percent was encountered in the non RSV cases (72%). Other studies have also reported excess antibiotic prescription during periods of RSV activity [29]; although numerous studies have shown that the occurrence of a secondary or concurrent bacterial infection in hospitalized children with RSV lower respiratory tract disease (LRTD) is <1% [30]. Unfortunately, this unjustified use of antibiotics increases the risk of development of antimicrobial resistance. These findings supports the imperative need for rapid reliable screening test to detect RSV infection to avoid unjustified use of antibiotics and also to reinforce implementation of infection control precautions among hospitalized cases, when diagnosis of RSV infection etiology is established.

IN CONCLUSION, RSV is the most common viral etiology of ALRTI in children below 5 years of age, especially among young infants less than 6 months of age. RSV infection was more often during winter months. Our results underscore the fact that routine testing children with ALRTI for RSV to avoid unnecessary antimicrobial therapy and to apply infection prevention and control precautions. Further studies are required to accurately define the risk groups who are in need for immunoprophylaxis.

## List of abbreviations

RSV: respiratory syncytial virus: ALRTI: acute lower respiratory tract infections: ED: Emergency Department: IFA: Immunoflourescent assay: FLU A/B: Influenza viruses' type A and B: PIV: parainfluenza virus: ADV: adenovirus: hMPV: human metapneumovirus: ELISA: enzyme linked immunosorbent assay: SPSS: Statistical Package for the Social Science: LRTD: lower respiratory tract disease.

## Competing interests

The authors declare that they have no competing interests.

## Authors' contributions

AF carried out patients' inclusion and examination, analysis of the data, interpretation of the results and drafting the manuscript. YM carried out patients' inclusion and examination, statistical analysis and interpretation of the results. MG performed the immunoassay. AA conceived of the study, participated in the design of the study and the immunoassay. HE conceived of the study, and participated in its design and coordination and helped to draft the manuscript. All authors read and approved the final manuscript.
